# Experiences of beginner runners on a modified Couch-to-5k programme: a qualitative exploration

**DOI:** 10.1093/heapro/daag131

**Published:** 2026-08-25

**Authors:** Nicola Relph, Danielle L Christian, Michael Owen

**Affiliations:** Health Research Institute, Faculty of Health, Social Care, and Medicine, Edge Hill University, Ormskirk, United Kingdom; Applied Health Research Hub (AHRh), University of Lancashire, Preston, United Kingdom; Health Research Institute, Faculty of Health, Social Care, and Medicine, Edge Hill University, Ormskirk, United Kingdom

**Keywords:** Couch to 5k, physical activity, running, qualitative, mental health, social support, adherence, musculoskeletal injury

## Abstract

Despite widespread recognition of the importance of physical activity in reducing the risk of chronic disease and high level of awareness and engagement with the Couch-to-5k programme, adherence to beginner running programmes remains low. Little is known about the experiences of ‘completers’ and effective approaches to improve and sustain engagement. This study explores the experiences of individuals completing a modified Couch-to-5k running programme, with particular focus on motivation, mental wellbeing, physical health, and injury perceptions. Grounded in an interpretivist paradigm, this qualitative study conducted five semi-structured focus groups with 33 participants who completed a 9-week programme incorporating weekly instructor-led group sessions alongside independent runs. Data were analysed using framework analysis. Four overarching themes were identified: programme motivation, mental health and wellbeing, perceived physical impact, and injury knowledge. Motivation evolved from individual health goals initially to a reliance on social support, with group-based, structured delivery enhancing accountability and adherence. Participants reported significant psychological benefits, including improved mood, self-efficacy, and social connectedness, alongside an emotional progression from anxiety to enjoyment. These benefits permeated wider cumulatively improving fitness, sleep, and health behaviours, despite initial discomfort and adaptation challenges. While no serious injuries were reported, minor issues highlighted the importance of pacing and body awareness, alongside gaps in injury-related knowledge. Findings suggest that socially supported, structured running programmes enhance adherence, while improving self-efficacy, physical health, and mental wellbeing. Integrating social components and injury education into future public health physical activity initiatives may improve engagement, reduce attrition, and promote sustained behaviour change.

Contribution to Health Promotion

- Socially supported, structured running programmes, such as the *Couch-to-5k*, can increase adherence to physical activity.
- Running programmes can deliver meaningful mental-health gains that strengthen public-health wellbeing strategies.
- Injury-education gaps highlight opportunities in health promotion to improve safety and sustain long-term physical activity engagement.
- Providing supportive environments enhance self-efficacy, a core driver of successful health-promotion interventions.

## Introduction

The importance of regular physical activity is well-recognized in both academic literature and approaches to public health as there is strong evidence of the dose-response relationship between it and chronic disease morbidity and mortality (Kesäniemi *et al*. 2010, O'Donovan *et al*. 2010, Warburton *et al*. 2010, Ekelund *et al*. 2019, Strain *et al*. 2020). Indeed, public health experts recognize ‘Exercise is Medicine’, with health professionals in the UK supporting the ‘Moving Medicine’ initiative to integrate physical activity conversations into routine clinical care (Faculty of Sport and Exercise Medicine 2018). Moreover, increased physical activity is a focus of England’s National Health Service (NHS) 10-year ‘sickness to prevention’ agenda, which plans to work with the Great Run Company to motivate millions to move more on a regular basis (NHS England 2025).

Inactivity increases the risk of major diseases such as Type II diabetes and cardiovascular disease (Lee *et al*. 2012), while the associated poor musculoskeletal (MSK) health accounts for the largest contribution to disability among non-communicable diseases (Briggs *et al*. 2023). Physical activity behaviour is also shaped by wider social and community influences, consistent with Dahlgren and Whitehead’s (Dahlgren and Whitehead 1991) model of the determinants of health, which situates individual lifestyle factors within layers of social and community networks and broader living conditions. To achieve good physical health the World Health Organization (WHO) recommends adults participate in 150 minutes of moderate-intensity aerobic physical activity or 75 minutes of vigorous-intensity aerobic physical activity, or an equivalent combination throughout the week (Bull *et al*. 2020). However, globally only one in four adults meet the recommended guidelines (Guthold *et al*. 2018) and physical inactivity is described as a ‘global pandemic’ (Hallal *et al*. 2012, Kohl *et al*. 2012, Lee *et al*. 2012). Yet, there are currently very little evidence-based recommendations which support the transition from inactivity to full recommended levels.

Public health initiatives have endorsed beginners running programmes to increase physical activity levels. For example, the NHS *Couch-to-5k* application is stated as being accessible, inclusive, simple, doable, and empowering to overcome barriers to exercise (Baber *et al*. 2021). Indeed, the Department of Health and Social Care in the UK reported over 8 million runs were completed using the application in 2023 (GOV.UK 2024). The benefits of completing the programme include reduced body mass index (BMI), increased fitness levels and physical activity maintenance (Stevinson *et al*. 2022, Relph *et al*. 2023a).

However, adherence to these programmes is low, ranging from 27.3% to 53%, even with an additional group support design (Stevinson *et al*. 2022, Relph *et al*. 2023b). Furthermore, non-completers of these programmes continue to have high BMI values and may not attempt physical activity again (Johnson *et al*. 2022, Relph *et al*. 2023b). This may be in part because population level adult physical activity initiatives are currently not evidence based. Indeed, current research suggests programmes can deter people from exercise all together, partly due to MSK injury occurrence and the demands of the running sessions (Relph *et al*. 2023b).

MSK injury rates of new and returning runners, are inconsistent, ranging between 7.5% (Nielsen *et al*. 2014) and 67% (Bovens *et al*. 2008); however, new runners are at a significantly higher risk of injury, up to 19% more likely, than recreational runners per 1000 hours of running (Videbæk *et al*. 2015). An understanding of injury incidence in new runners is important as previously stated, experience of a MSK injury during physical activity can be a deterrent from future engagement (Relph *et al*. 2023b). For example, 78% of those injured during a running programme were still absent after six weeks (Smits *et al*. 2016). Furthermore, 40% of women and 37% of men did not re-start running after injury and this pattern was higher in novice runners (48%) than those already engaged in running (24%) (Buist 2008). Any injury is likely to reduce current physical activity levels (Andrew *et al*. 2014); however, evidence suggests new runners may be permanently deterred from future physical activity following an injury (Relph *et al*. 2023b).

There is currently limited evidence on the experiences of those who complete a beginner running programme, such as the *Couch-to-5k*. With high drop-out rates in physical activity initiatives, this information is critical to understanding the reasons for success in running programmes to help better design and support of public health programmes in the future. The Couch-to-5k programme also offers a distinctive research context. It is a nationally endorsed, free, app-supported public health intervention with exceptionally high public awareness and reach, delivered through a standardized nine-week progression. Understanding what supports completion of this specific programme, rather than exercise initiation in general, can therefore directly inform the design and delivery of an intervention already operating at national scale. Therefore, the aims of this study are to explore:

1. To explore the thoughts, perceptions, and motivations behind completing a modified *Couch-to-5k* programme.
2. To explore the perceived impact of the modified *Couch-to-5k* programme on participants mental health and wellbeing.
3. To explore the perceived physical impact of the modified *Couch-to-5k* programme.
4. If an injury occurred during the programme, explore knowledge on injury prevention and/or treatment and associated link to participant’s mental health and wellbeing.

## Materials and methods

### Research paradigm and study design

This study was grounded in the interpretivist paradigm, which assumes that social phenomena, such as the meanings individuals attach to exercise and physical activity, are socially constructed and best understood through participants’ subjective experiences (Lincoln 1985, Willis 2007). Rather than seeking generalizable truths, the research aimed to explore how individuals interpret and negotiate meaning within their social contexts. The interpretivist stance emphasizes co-creation of knowledge, where understanding emerges through interaction between researcher and participants (Schwandt 2000, Willis 2007). Accordingly, while the study did not seek statistical generalizability, the recommendations can be made on the basis of transferability; rich contextual description of the programme, participants, and setting is provided so that readers can judge the applicability of the findings to comparable programmes and populations (Lincoln 1985).

To capture these shared interpretations of the programme, we employed a focus group design. Focus groups are particularly suited to interpretivist inquiry because they enable participants to articulate and refine their views through dialogue, revealing both individual and collective perspectives (Amir *et al*. 2024). While individual interviews or surveys could also have captured personal motivations, perceptions, and injury experiences, focus groups were selected because the modified programme was itself experienced collectively: participants trained together each week, and discussion among those who had shared the group sessions allowed experiences of the programme’s social and structural elements to be compared, challenged, and refined in interaction. This was considered important given that the social dimension of the modified delivery was a central focus of the study. In this study, the researchers (M.O. and N.R.) acted as facilitators, encouraging open discussion while allowing participants to shape the conversation organically. This approach provided rich, contextual data on participants lived experiences and the meanings they attribute to them. By situating the research within an interpretivist framework and using focus groups as the primary method, the study was able to generate nuanced insights into the social processes underpinning participants’ interpretations, consistent with the paradigm’s emphasis on depth, context, and subjectivity.

### Participants

Participants were recruited from Northwest England during attendance at a voluntary induction session where the lead researcher introduced and explained the study. Any person who completed the final Couch-to-5k session was invited to a focus group. In total 33 participants (84.6% of all completers, males = 7 (21.2%), and females = 27 (81.8%)) volunteered to take part in the study. Demographic data beyond sex, such as age, were not formally collected.

The 9-week running programme consisted of 3 sessions a week with run time increasing incrementally (see Table 1) following the NHS England guidelines (NHS.uk 2025). However, the first of these sessions each week was instructor-led at a local running track and included community and individual level social support to increase retention and engagement as per learning in physical activity interventions (Terry and Hogg 1996; Heath et al. 2012; Strachan et al. 2012; Stevens et al. 2019). The remaining two runs each week were self-led. This weekly instructor-led group session, in place of one of the standard self-led runs, was the sole modification to the NHS Couch-to-5k programme; the run durations and weekly progression were otherwise unchanged (Table 1).

**Table 1:** The modified delivery of Couch-to-5k, including the one instructor-led run each week, based on (NHS.uk, 2025).

	Run 1 (instructor-led)	Run 2 (self-led)	Run 3 (self-led)
Week 1	Brisk 5-min walk, then 1-min run + 1.5-min walk × 8	Brisk 5-min walk, then 1-min run + 1.5-min walk × 8	Brisk 5-min walk, then 1-min run + 1.5-min walk × 8
Week 2	Brisk 5-min walk, then 1.5-min run + 2-min walk × 6	Brisk 5-min walk, then 1.5-min run + 2-min walk × 6	Brisk 5-min walk, then 1.5-min run + 2-min walk × 6
Week 3	Brisk 5-min walk, then 1.5-min run + 1.5-min walk + 3-min run + 3-min walk × 2	Brisk 5-min walk, then 1.5-min run + 1.5-min walk + 3-min run + 3-min walk × 2	Brisk 5-min walk, then 1.5-min run + 1.5-min walk + 3-min run + 3-min walk × 2
Week 4	Brisk 5-min walk, then 3-min run + 1.5-min walk + 5-min run + 2.5-min walk + 3-min run + 1.5-min walk + 5-min run	Brisk 5-min walk, then 3-min run + 1.5-min walk + 5-min run + 2.5-min walk + 3-min run + 1.5-min walk + 5-min run	Brisk 5-min walk, then 3-min run + 1.5-min walk + 5-min run + 2.5-min walk + 3-min run + 1.5-min walk + 5-min run
Week 5	Brisk 5-min walk, then 5-min run + 3-min walk + 5-min run + 3-min walk + 5-min run	Brisk 5-min walk, then 8-min run + 5-min walk + 8-min run	Brisk 5-min walk, then 20-min run
Week 6	Brisk 5-min walk, then 5-min run + 3-min walk + 8-min run + 3-min walk + 5-min run	Brisk 5-min walk, then 10-min run + 3-min walk + 10-min run	Brisk 5-min walk, then 25-min run
Week 7	Brisk 5-min walk, then 25-min run	Brisk 5-min walk, then 25-min run	Brisk 5-min walk, then 25-min run
Week 8	Brisk 5-min walk, then 28-min run	Brisk 5-min walk, then 28-min run	Brisk 5-min walk, then 28-min run
Week 9	Brisk 5-min walk, then 30-min run	Brisk 5-min walk, then 30-min run	Brisk 5-min walk, then 30-min run

### Data collection

Five focus groups were conducted using a semi-structured focus group approach. The focus group questions explored motivations for taking part in the programmes, perceptions on the impact of the programme on physical health, mental health and wellbeing, and the experiences of any injuries. Focus groups lasted an average of 40 minutes (ranging from 22 to 69 minutes) with an average of six and seven participants per group. The study was conducted according to the guidelines of the Declaration of Helsinki and approved by the Ethics Committee of Edge Hill University (Ref no. HW3, 1 May 2018). Written informed consent was obtained from all subjects involved in the study.

### Data analysis

Qualitative data from the focus groups were analysed using the Framework Method (Gale *et al*. 2013). This approach provides a systematic and flexible model for managing and mapping qualitative data, specifically through a matrix-based output that allows for comparison across cases and codes while maintaining the context of individual accounts. The analysis followed the seven stages described by Gale *et al*. (2013):

1. Transcription: Audio recordings were transcribed verbatim to provide a high-quality textual record, focusing on the content of the dialogue.
2. Familiarization: Researchers (M.O. and D.C.) immersed themselves in the data by reading transcripts and reviewing reflective field notes to identify early analytical impressions.
3. Coding: Initial open coding was conducted independently on the first few transcripts, applying descriptive or conceptual labels to passages to identify substantive ideas, values, and emotions.
4. Developing a Working Analytical Framework: After independent coding, the researchers met to compare their labels and agree on a working analytical framework, a set of codes organized into categories (linked to research questions) used to manage the data.
5. Applying the Analytical Framework: This framework was then used for indexing, which involved the systematic application of the agreed codes and categories to the remainder of the dataset.
6. Charting Data into the Framework Matrix: Data were reduced and charted into a matrix, a spreadsheet containing rows (representing individual focus groups/cases) and columns (representing codes/categories). This allowed for the summarization of data while retaining the ‘feel’ of the participants’ original words and specific illustrative quotations.
7. Interpreting the Data: In the final stage, the research team interrogated the matrix to identify characteristics and differences between cases, mapping connections between categories to develop interpretive themes that explain aspects of the data.

Throughout this process, researchers maintained reflexivity. This was achieved by recording impressions in analytic memos and engaging in critical dialogue to ensure that the findings were rigorous, transparent, and provided a clear audit trail from the raw data to the final themes. All codes and emergent themes were checked between the two authors (M.O. and D.C.) who conducted the analysis to ensure consistency of coding. Any disagreements were discussed until an agreement was reached. After finalizing themes, quotes that were deemed to best represent each theme were then selected to illustrate the wider views of the participants. To ensure methodological rigor, credibility, and trustworthiness (Nowell *et al*. 2017, Levitt *et al*. 2018), the final themes were then cross-examined against the data in reverse (M.O. and D.C.), from the themes to the data sheets.

## Results

Five focus groups were conducted with 33 participants. Framework analysis generated four overarching themes: Programme Motivation, Impact on Mental Health and Wellbeing, Perceived Physical Impact of the Programme, and Injury Knowledge and Experiences.

### Programme motivation

Participants’ decisions to undertake the modified *Couch-to-5k* programme were driven by internal and external influences, while their experiences of the programme itself were shaped by its structural design and delivery approach. Three sub-themes were generated within this theme: personal motivations, external influences and social support, and perceptions of the programme structure. In line with self-determination theory’s distinction between motivation and the social-contextual factors that support it (Deci and Ryan 2000), participants’ accounts of encouragement from others are framed here as external influences and social support rather than as motivation *per se*.

### Personal motivations

Participants were primarily motivated by personal health and fitness aspirations. With one participant noting upon completion, ‘After the last ten weeks, I know I’ve lost the weight and that was a goal as well, but the goal was to do what I did tonight and do the 5k, but it’s improved the feeling of the whole body’ (FG3, P2). This internal drive was linked to specific life stage considerations, particularly among participants approaching significant age milestones. As one participant explained, ‘50 is looming you know and I’ve got friends who were pretty active, and I felt like I was the one that wasn’t’ (FG1, P2). These internal motivation factors seemed to encourage participants to begin the programme, and then individual goals supported them to complete the programme.

### External influences and social support

Social influences played an important role, with friends, family members, and peer networks providing both encouragement and accountability.

‘I’ve had a lot of support from my family and my husband has been very supportive.’ (FG2, P7).

‘Mine (husband) even came to check that this is what I was doing on a Monday night…[laughing]…I came round the bend, and I went, there’s [P8 husband], yes I am running.’ (FG2, P8).

Participants consistently highlighted the significance of social support in physical activity adherence. Seasonal considerations also influenced participants’ decisions to begin the programme. Many deliberately timed their start to coincide with favourable weather conditions, recognizing the psychological and practical benefits of beginning during spring or summer months. As one participant noted, ‘That’s why I did it first time in the spring/summer time and that was really nice because you could go out and train and the difference in the winter, I just think, I find it easier in the summer than in the winter months’ (FG2, P6).

### Perceptions of programme structure

Participants’ experiences of the group-based run element of the modified programme were positive, with appreciation for its structural elements. The programme was perceived as accessible, offering a structured pathway into running, and the guidance and encouragement provided by instructors throughout was identified as a valued element of its delivery. The programme's modified framework was valued, with many participants expressing relief at having explicit guidance rather than having to design their own training regimen. As one participant explained, ‘each week and you have kind of got to know us all, our little ways and what have you, you keep on supporting us and it’s really well organised, and communication is good in terms of what’s going to be happening, and any changes’ (FG1, P2).

There was a gradual progression to the running programme noted by participants, which they valued, but some noted big jumps in distance at certain points in the programme. For example, in week 4 participants are asked to run continually for 5-minute blocks, however, at week 5 the runs jump from 5-minutes to 8-minutes to 20-minutes blocks. Similarly, the flexibility of completing the sessions when they had time was important to many participants. The self-paced nature of the programme allowed participants to listen to their body and complete the programme at their own pace and the instructors gave them to confidence to do this.

The group-based delivery proved critical for programme completion, with participants consistently identifying its importance.

‘I think it really did well with the social aspect of it, looking forward to asking everyone when they were coming on a Monday, how did you do? and it’s nice to catch up.’ (FG2, P4)

‘I tried to do Couch-to-5k by myself and it didn’t really work, I found doing it with people was much better and having motivation inside of you, you’ve got to do it because you’re doing it with other people type of thing. I thought it was really helpful.’ (FG2, P5).

This collective approach provided essential support structures that enhanced adherence and psychological comfort, with participants noting they felt supported. The non-competitive group environment particularly benefited mental health and wellbeing outcomes, creating conditions where participants felt psychologically safe to challenge themselves physically. However, this group dependency created challenges for runs outside of the group sessions and after completing the programme. Participants noted struggles with motivation maintenance after programme completion due to not having weekly sessions as a group.

### Impact on mental health and wellbeing

The modified *Couch-to-5k* programme demonstrated positive effects on participants’ mental health and wellbeing, extending beyond the physical benefits of regular exercise. The psychological impact linked to three sub-themes: psychological benefits, emotional journey, and social wellbeing.

### Psychological benefits

Participants consistently reported significant improvements in their overall mood and emotional regulation following engagement with the programme. These changes were often noticed in everyday contexts, with participants describing how they became more patient with kids and have a better overall mood.

‘In my head, I do feel better because you feel a bit more positive and there’s something to look forward to’ (FG5, P4).

‘I have much more patience for my munchkins (kids) yes’ (FG1, P1).

The mood-enhancing effects appeared to extend beyond the immediate post-exercise period, suggesting potential lasting psychological benefits.

The running experience also provided participants with enhanced mental clarity and opportunities for mindfulness. Many described their running sessions as providing mental space and cognitive respite from daily stressors. As one participant explained,

‘I’ve got a local field as well, like a football pitch, so I run down, it’s a nice little stretch in the local village and then you know, a few laps of the actually football field and it’s kinda nice, you know mindful.’ (FG1, P1). This mental clearing effect appeared particularly pronounced when participants ran in natural environments, suggesting the additional psychological benefits of outdoor exercise.

Participants frequently expressed surprise at their own capabilities, with many experiencing a shift in self-perception. The sense of accomplishment derived from completing the programme was evident in comments such as ‘I think with me it was something that I thought I could never do…because I’d never done it before and I couldn’t do it…and that proved to myself that I can do… I’ve enjoyed it’. This evidenced enhanced self-belief and confidence. Although motivated to start, many participants began the programme with self-doubt about their running capabilities. There was a shift from self-doubt to self-efficacy, which developed as an outcome of the programme experience.

The group-based nature of the programme also appeared to contribute to reduced self-consciousness around physical activity. Many participants noted feeling less inhibited in their running efforts, with the non-competitive environment fostering psychological comfort. As participants described the group environment: ‘it’s just comfortable really. And nobody was saying, you know, we were all new to it, it was fine, it didn’t matter where you were at, and you didn’t feel self-conscious in any way and I think that makes a big difference’ (FG1, P3), indicating that the supportive group context was important for psychological wellbeing.

### Emotional journey

Participants were initially anxious and apprehensive when starting the programme. Many participants were fearful of running before the programme started, but once they started attending sessions this anxiety reduced. The early negative emotions appeared to be normal and expected aspects of beginning a new physical challenge.

As participants progressed through the programme, these initial anxieties were gradually replaced by feelings of achievement and pleasant surprise at their own progress. The emotional shift was evident in participants’ descriptions of their journey through the programme:

‘I’ve learnt to love running again. When I went out on my own at the weekend, I did 3k, but I loved it and I just thought ‘Isn’t it amazing? The body is an amazing thing and I’ve got the ability to do it, so, you know, why not do it? Why stand on the side-lines and watch other people achieve it, you know?’ So, this is my journey. My fitness journey and my goal and…I’m loving it.’ (FG3, P3)

‘It is good when you’re improving on that, you’re getting longer and longer each week and you don’t really think that you’re going to be able to do it to begin with and to know that you’ve done it is a good achievement’ (FG2, P7)

‘It is a great feeling once you’ve done it, you’ve achieved that and you think, wow, yeah I have done it, good, great.’ (FG2, P1)

This transformation from trepidation to enjoyment appeared to be a key factor in maintaining programme engagement. However, the emotional journey was not uniformly positive, with participants experiencing fluctuations in motivation throughout the programme duration. Some commented on the difficulty of doing runs on their own. These motivational challenges highlighted the importance of ongoing support and the difficulty of transitioning from group-based to independent running.

Despite successfully completing the modified *Couch-to-5k* programme and maintaining regular running routines for the duration of the programme, many participants continued to view themselves as ‘joggers’ rather than ‘runners’.

‘I wouldn’t call that running. I’d call it jogging.’ (FG4, P3).

‘I look at those (runners) and that’s what I see as being someone who you know, is physically fit, does a bit of exercise, runs and all that kind of stuff. Erm…but it’s not necessarily what I would picture myself as.’ (FG 5 P4).

This choice of language may tentatively indicate that some participants did not yet view themselves as ‘runners’, despite running two to three times per week, and that psychological identity change can lag behavioural change. However, this interpretation rests largely on participants’ choice of words.

### Social wellbeing

The group-based delivery of the modified *Couch-to-5k* programme generated significant social wellbeing benefits that were integral to participants’ overall positive experience. Enhanced social connections were identified as a primary benefit, with many participants valuing the opportunity to run together in a group of similar level peers.

The sense of group belonging fostered by the programme was consistently identified as a key factor in participants’ positive experiences. Being part of a cohort of individuals at similar fitness levels and life stages created a supportive environment that enhanced psychological comfort. Participants appreciated that group environment; ‘it’s the group…because it’s…people are motivating and inspiring others’ (FG3, P6) and everyone in the group being a similar level as important to reduce comparison-based anxiety and foster mutual encouragement.

These findings demonstrate that the modified *Couch-to-5k* programme's impact on mental health and wellbeing was multifaceted, encompassing direct psychological benefits, emotional transformation, and enhanced social connection. The interconnected nature of these domains suggests that the programme's group-based approach was fundamental to its psychological effectiveness, creating conditions that supported both individual growth and social wellbeing.

### Perceived physical impact of the programme

The modified *Couch-to-5k* programme impacted physical transformations that extended beyond basic fitness improvements to encompass broader health awareness and lifestyle changes. The perceived physical impact of the programme linked to two sub-themes: positive physical changes and physical challenges and adaptions.

### Positive physical changes

Participants consistently reported the programme successfully enhanced their aerobic capacity and overall cardiovascular health. The programme also fostered enhanced body awareness, with participants becoming more attuned to their physical needs and responses. For participants with pre-existing health conditions, the programme provided unexpected physical breakthroughs. Those with asthma particularly valued the opportunity to challenge previous limitations.

‘I hated running, I thought I’d never be able to run. I have got asthma. And I always thought it would just stop me from doing all sports so I just never bothered really to do anything that was strenuous. So I am amazed I’ve got in to this.’ (FG1, P1)

These achievements represented significant victories over perceived physical constraints. Similarly, these perceived physical benefits extended to broader lifestyle improvements, with participants reporting better sleep quality, weight management, and adherence to other health behaviours. This suggests that running success positively impacted changes across multiple health domains.

‘I’m doing Slimming World as well but I think it’s because…I feel as though I’m eating healthier because I don’t want the junk because it just makes me feel so sluggish and awful…and I want the energy to be able to do this.’ (FG4, PN4).

‘I think I’m tireder[sic] because obviously I’m doing exercise so it gets to sort of like ten o’clock at night and I really do want to go to bed…whereas I’ve always been quite a night owl. So now I’m ready for bed at ten and I’m probably nodding off quite quickly and very annoyingly waking up at about half past five instead now.’ (FG4, PN3).

### Physical challenges and adaptations

Initial physical discomfort to running was reported by nearly all participants including tiredness, shortness of breath, and painful legs. This early discomfort was accompanied by adaptation challenges, including needing rest days.

‘Initially, I think I was very tired after a run. So initially I needed that day’s rest. Definitely I needed a day’s rest. In fact, it was hard sometimes to go and do the Wednesdays run, I was still tired from the Monday initially’ (FG1, P2).

Environmental factors also presented ongoing challenges, with participants noting that the weather can impact their ability to run in summer and in winter. However, participants developed effective strategies to manage these challenges. For example, pacing was identified as a crucial skill:

‘I’d never run as fast as I could, coz [sic] I don’t think like mentally I could do that. I’d just go at a pace like, I was like oh I can still breathe, I can do this. And if I needed to ease off I would. And I was like just try and find that place where I was comfortable…[unclear]…but I think like you were saying about the stop start, you want to fire off and like you only run for a minute it’s like you want to sprint. But that’s not really how you are supposed to run.’ (FG1, P3).

Participants reported the importance of running at their own pace, with some running ‘very slowly’ in order to avoid injury. These findings demonstrate that while physical challenges were inevitable, participants successfully developed resilience and adaptive strategies that enabled programme completion and ongoing running engagement.

### Injury knowledge and experiences

While no participants reported serious injuries, their experiences revealed important insights into injury prevention strategies, minor physical issues, and the psychological dimensions of injury-related concerns. The injury knowledge and experiences were linked to two sub-themes: injury prevention and management approaches and knowledge gaps and psychological impact.

### Injury prevention and management approaches

Participants developed effective injury prevention strategies through the programme, primarily through cautious pacing and heightened body awareness. Many adopted conservative approaches, with participants noting they needed to listen to their bodies for each run. This self-regulation appeared crucial for preventing more serious injuries.

‘I could feel it twinging a bit. So I just slowed down a bit, now it’s fine’ (FG1, P8)

Minor issues were common, including shin pains after longer runs, sore knees, and overall shin pain. Participants managed these through basic injury management approaches including reducing load, and hot and cold therapy.

### Knowledge gaps and psychological impact

Despite successful self-management, participants identified knowledge gaps. Many expressed desire for more guidance and information at the start of programme on how to run and information on pacing.

‘It might be a good idea to do something like that part way through the course; maybe at the beginning on how to run, your arm movements and your breathing and foot placements’ (FG2, P6)

This suggests that more comprehensive education on running technique and injury prevention could enhance programme effectiveness. Moreover, fear of injury influenced participants’ confidence and approach, with some noting injury fears affecting confidence and nervousness about progressions. However, successful prevention strategies enhanced confidence, with participants reporting that new trainers and self-paced approaches were beneficial. These findings highlight the interconnected nature of physical injury prevention and psychological wellbeing within the programme experience.

## Discussion

Five focus groups explored the thoughts, perceptions, and motivations behind completing a modified *Couch-to-5k* programme and the impacts on physical health, mental health, and injuries. Programme Motivation, Impact on Mental Health and Wellbeing, Perceived Physical Impact of the Programme, and Injury Knowledge and Experiences were all identified as main themes salient to answering the research aims. While several of these findings echo the broader physical activity and exercise adherence literature, the study makes several contributions that are specific to, and generated by, this research. To our knowledge, it is the first qualitative exploration of completers of a modified, group-supported *Couch-to-5k* programme, and it offers four novel insights: motivation evolved across the programme, from individual health goals at the outset to reliance on the group and its social support for completion; the standardized progression, although valued, contained perceived jumps in demand (notably at week 5) that programme designers could address; runner identity lagged behind behavioural change, with completers still describing themselves as ‘joggers’; and completers wanted education on running technique, pacing, and injury prevention embedded within the programme. These insights extend an evidence base that has largely focused on adherence rates and beginner runners in general, rather than on those who complete this widely used public health programme.

### Programme motivation

Our findings indicate that motivation to complete the programme evolved over time. While initial engagement was primarily driven by individual goals such as weight loss and improved fitness, sustained participation appeared to be strongly influenced by the modified delivery format and the social support embedded within the group-based approach. This observation aligns with evidence that social support is a critical determinant of exercise adherence, with group-based interventions consistently outperforming individual programmes in terms of retention and behavioural persistence (Burke *et al*. 2006).

The original *Couch-to-5k* programme was typically designed for solitary completion, which may limit opportunities for peer interaction and shared accountability. In contrast, the current study suggests that incorporating structured social elements, such as group runs and peer encouragement can enhance motivation and reduce attrition. This is supported by research demonstrating that group cohesion and interpersonal support foster positive achievement-related emotions, which in turn promote sustained engagement in physical activity (Zhang *et al*. 2025). Furthermore, meta-analytic evidence indicates that interventions delivered in ‘true groups’, where social bonds and collective identity are cultivated, yield significantly greater adherence compared with home-based or minimally interactive formats (Burke *et al*. 2006).

These findings underscore the importance of embedding social support mechanisms into public health initiatives aimed at increasing physical activity. Group-based interventions not only provide accountability but also foster a sense of belonging and shared identity, which are critical for sustaining motivation over time. Evidence from social identity theory suggests that when individuals perceive themselves as part of a valued group, their commitment to group norms, such as regular exercise, strengthens significantly (Carron and Spink 1993). More recent meta-analytic work confirms this, demonstrating that internalizing physical activity roles and group memberships is strongly associated with participation, with role identity showing a correlation of *r* = .40 across 35 studies and social identity *r* = .20 across 15 studies (Liddelow *et al*. 2025). These findings highlight that identity processes are not peripheral but central to behaviour maintenance.

Furthermore, interventions that actively cultivate group cohesion and peer support have been shown to amplify both psychological and behavioural outcomes compared with individual approaches (Burke *et al*. 2006). This suggests that future programmes should integrate structured social components, such as team-building activities, peer mentoring, and identity-affirming milestones, to optimize engagement and long-term adherence. Future versions of the *Couch-to-5k* programme could also consider integrating opportunities for social interaction within the online app platform. Embedding these strategies within public health initiatives could transform exercise from an isolated task into a socially meaningful experience, thereby enhancing engagement and sustainability. These findings also resonate with Dahlgren and Whitehead’s 1991 model of the determinants of health, illustrating how the social and community networks layer of that model can be actively mobilized within public health programmes to support individual behaviour change (Dahlgren and Whitehead 1991).

### Impact on mental health and wellbeing

The modified C25K programme produced notable psychological benefits, including participants reporting improved mood, emotional regulation, and mental clarity. These findings align with evidence that physical activity significantly improves mental health across diverse populations (Schuch *et al*. 2018). Even modest amounts of activity have been associated with lower risk of depression, reinforcing the mental health value of accessible programmes (Schuch *et al*. 2018). Participants’ descriptions of ‘mental space’ and mindfulness during outdoor runs in green space corresponds with research on ‘green exercise’, which demonstrates that exposure to natural environments enhances mood and self-esteem beyond the effects of exercise alone (Barton and Pretty 2010). These results suggest that the programme’s outdoor, progressive design may have amplified its psychological impact.

Participants’ emotional journey from initial anxiety to enjoyment and accomplishment reflects the role of mastery experiences in building self-efficacy (Bandura 1997). This progression is consistent with Self-Determination Theory, which emphasizes competence and autonomy as predictors of sustained motivation (Deci and Ryan 2000, Teixeira *et al*. 2012). While early apprehension diminished over time, identity transformation appeared to lag behind behavioural change, with many continuing to identify as ‘joggers’ rather than ‘runners’. This suggests that psychological identity may require sustained reinforcement beyond programme completion. Interventions that explicitly support identity development may therefore enhance long-term adherence.

The group-based structure of the programme was identified as a critical factor in promoting social wellbeing and reducing self-consciousness. This finding is supported by research linking group cohesion to improved adherence and satisfaction in exercise settings (Carron and Spink 1993, Graupensperger *et al*. 2019, Beauchamp *et al*. 2021). Community-based initiatives such as parkrun similarly demonstrate that social ties foster motivation and mental wellbeing (Stevinson *et al*. 2022). Signposting completers towards parkrun or other social running groups at the end of the programme may therefore offer a means of continuing the social benefits of running identified in this study; indeed, Couch-to-5k participants who graduate to parkrun report continued physical activity maintenance (Relph *et al*. 2023a). However, participants’ motivational dips when transitioning to independent running reflect a common challenge in behaviour maintenance. Theoretical reviews highlight the need for ongoing social and environmental support to sustain behaviour change beyond initial adoption (Kwasnicka *et al*. 2016). Future programmes should consider structured post-intervention strategies, such as buddy systems or identity-affirming milestones, to maintain engagement and promote social wellbeing. These findings reinforce the importance of social support in exercise interventions, suggesting that community-oriented approaches may amplify psychological benefits and improve retention compared with solitary programmes.

### Perceived physical impact of the programme

Completers of the modified *Couch-to-5k* programme reported positive physical changes, including self-reported increases in fitness and improved sleep, diet, and body and health awareness. There is limited previous evidence on the *Couch-to-5k* programme specifically, but our findings are aligned with other evidence that links physical activity with perceived improvements in physical health. For example, qualitative studies examining running maintenance have shown that new runners value tangible benefits, such as improved sleep and weight loss (McCormick *et al*. 2024). New runners have also reported an increase in perceived competence (Johnson *et al*. 2022) and a reduction in social physique anxiety (Plateau *et al*. 2024) following a similar beginner runner programme to the *Couch-to-5k* programme, which may be linked to improved fitness and weight. The perception of an improvement in fitness was also reported by those in the current study with long-term conditions such as asthma. This may explain why participants were able to complete the programme, as they had a personal and meaningful reason for running (McCormick *et al*. 2024, Jung *et al*. 2025).

Completers of the modified *Couch-to-5k* programme also discussed the physical challenges of the running programme such as tiredness, shortness of breath, and painful legs reported by almost all participants. This identifies an area for physical activity practitioners to consider in the future. Initiatives may require embedded MSK health education, including information on delayed onset muscle soreness, so people are aware of how their body may feel once beginning. Indeed it has been previously stated that the adverse effects of physical activity need to be more readily collected and reported in research, not to present a barrier but to improve education (Verhagen *et al*. 2015). The participants in the current study reported overcoming these physical challenges and adapting to running, such as improving pacing strategies, was a key positive reflection of the programme. Therefore, pacing education should also be included in beginner running programmes.

### Injury knowledge and experiences

Although all participants completed the programme, some reported experiences of injury and lower limb pain during the programme. A positive response to this was an increase in body awareness which allowed participants to find their own strategies to self-regulate running speed and reduce the pain. Reducing speed and hence joint loading is a well-recognized strategy to reduce injury risk (Choe *et al*. 2021). However, previous evidence suggests new runners or exercisers may adopt a ‘play now, pay later’ strategy embedded in a culture of risk and performance focus (Grice *et al*. 2014, Malcolm and Pullen 2020, Sicilia *et al*. 2026). New runners may be unaware of the long-term consequences of continuing activity when injured and subsequently fail to seek medical advice (Grice *et al*. 2014). Furthermore, new exercisers who experience an injury may become more motivated by external standards such as specific running distances that they cannot achieve, which leads to frustrations and negative feelings towards running (Sicilia *et al*. 2026). For example, an injured runner may continue the *Couch-to-5k* programme because of the time limited design, feeling pressured to run when injured. Therefore, beginner running programmes should include information on injury prevention, treatment, and management, including the potential long-term impact of injury.

### Strengths and limitations

This study offers a unique insight into the motivations and facilitators aiding completion of a modified *Couch-to-5k* programme. There is a possibility of selection bias with those who viewed the programme more positively more likely to participate in the focus groups. However, 84.6% of all completers participated in the focus groups suggesting these findings are representative of those who would typically complete the programme. Similarly, while there were more female than male focus group participants, this was representative of the demographics for all completers. However, demographic data beyond sex, such as participant age, were not collected, which limits the description of the sample and the contextualization of the findings.

A further limitation is that the study captured only the experiences of programme completers and therefore carries an inherent ‘success story’ bias, as the perspectives of those who did not complete the programme, who are arguably the primary target of health promotion efforts, were not represented within this study. However, the experiences of non-completers have been examined in the research team’s earlier work, in which interviews with drop-outs from the same modified Couch-to-5k programme identified MSK injury, negative emotions linked to non-completion, and the progressive design of the programme as key factors in attrition (Relph *et al*. 2023b). The present study complements that work by explaining what supports completion, although the facilitators identified here cannot be assumed to fully address the barriers experienced by those who drop out, and further research with larger samples of non-completers would strengthen understanding of attrition from beginner running programmes.

Individual adherence to the full *Couch-to-5k* programme was not closely monitored so it is difficult to evaluate fidelity, though this was a purposeful approach to prevent influencing typical practice and to more effectively reflect participation in a real-world situation.

Focus groups were undertaken immediately after the last session, increasing memory recall and validation of responses in the short-term. Though further research exploring any longer-term effects would be warranted.

## Conclusion

The *Couch-to-5k* public health initiative is widely known, has a high level of engagement, and has been shown to have positive physical and mental health benefits. Participants in this study who completed a modified version of *Couch-to-5k* reported additional cumulative health behaviour changes, over and above physical activity alone, including positive changes to diet, sleep and psychological wellbeing. Social support improved initial engagement with the programme and encouraged sustained participation throughout. Future public health running programmes should consider incorporating community networking either with in-person running groups (such as graduation to parkrun) or digitally within online forums or applications for social support and further information on running style, breathing techniques, and injury prevention.

## Data Availability

Data not available—participant consent. The participants of this study did not give written consent for their data to be shared publicly, so due to the sensitive nature of the research supporting data is not available.
